# Plasmidial virulence of *Rhodococcus equi* and its implications of livestock infections for human health: a possible foodborne pathogen?

**DOI:** 10.1590/S1678-9946202668023

**Published:** 2026-03-13

**Authors:** Márcio Garcia Ribeiro, Alexandre Naime Barbosa, Juliano Gonçalves Pereira, Fábio Sossai Possebon, José Paes de Almeida Nogueira Pinto, Fábio Vinícius Ramos Portilho, Thaís Spessotto Bello, Patrik Júnior de Lima Paz, Marcelo Fagali Arabe, Letícia Colin Panegossi, Alec Utida Reznik, Larissa Onuki Zeferino, Maria Clara Trindade, Nícolas Garcia Ribeiro, Shinji Takai

**Affiliations:** 1 Universidade Estadual Paulista, Faculdade de Medicina Veterinária e Zootecnia, Departamento de Produção Animal e Medicina Veterinária Preventiva, Botucatu, São Paulo, Brazil; 2 Universidade Estadual Paulista, Faculdade de Medicina, Departamento de Infectologia, Dermatologia, Diagnóstico por Imagem e Radioterapia, Botucatu, São Paulo, Brazil; 3 Fundação Educacional do Município de Assis, Assis, São Paulo, Brazil; 4 Kitasato University, School of Veterinary Medicine and Animal Sciences, Department of Animal Hygiene, Towada, Aomori, Japan

**Keywords:** pVAP types, Rhodococcosis, Epidemiology, One Health, Overview

## Abstract

*Rhodococcus equi*, a versatile and adapted opportunistic in nature bacterium, infects animals and humans. This soil-borne microorganism widely occurs in farms. Its dissemination occurs by feces from domestic animals (particularly horses, cattle, and pigs). In the last decades, bacterial virulence has been strongly attributed to plasmid-encoded virulence-associated proteins (VAPs). To date, three virulence plasmid types have been recognized: pVAPA, pVAPB, and pVAPN, which are considered host- or livestock-adapted. The pVAPA type is related to equine isolates (horse-type), the pVAPB type is associated with pig isolates (porcine-type), whereas the pVAPN type occurs in domestic ruminants (bovine and caprine) (ruminant-type). Nonetheless, pathogenic *R. equi* possessing the three virulent plasmid types can infect humans. Inhaling aerosol particles from the environment of equids represents the first route traditionally considered for the transmission of *R. equi* to humans, although an epidemiological lack of transmission remains in human infections because some patients with rhodococcosis have no history of contact with livestock or their environment on farms. However, all pVAPs types have been found in humans infected by *R. equi* (predominantly living with HIV), which could be presumably transmitted to patients by the ingestion of contaminated undercooked or raw meat from slaughtered pigs, cattle and, occasionally, horses, representing a probably route of the transmission of the pathogen from livestock-to-humans that could partially explain infections in humans without a history of contact with cattle, pigs, horses, or their farm environments.

## INTRODUCTION

### Foodborne pathogens

Some microorganisms play a relevant role in food production, safety, and spoilage. Nonetheless, the interactions of microorganisms with foods can make them inappropriate for human consumption because a variety of these organisms are notoriously versatile, adapted, primary, or opportunistic pathogens in nature due to a set of virulence factors showing risks to food safety and illness for human health worldwide^1^.

Foodborne diseases constitute major threats to human society, requiring one health approaches. The World Health Organization has estimated that 600 million (almost 1 in 10 people worldwide) will fall ill after eating contaminated food. Each year, an estimated 420,000 people die from contaminated food (particularly children aged less than five years), 125,000 of whom die/year from foodborne diseases, which is considered a high risk of mortality^2^.

Globally, a set of microorganisms, estimated to include more than 200 pathogens, are related to foodborne case-reports or outbreaks, including bacteria, viruses, fungi, parasites, and protozoa^1-3^. *Staphylococcus aureus, Bacillus cereus*, *Clostridium perfringens, Clostridium botulinum, Listeria monocytogenes*, some species of *Campylobacter, Vibrio*, and *Shigella*, as well as specific serovars of *Escherichia coli* and *Salmonella* are among the most common reported bacteria related to foodborne outbreaks^1^. Moreover, norovirus, rotavirus, and hepatitis A^3^ and E^4^ have been described as foodborne pathogens for humans, whereas some species of fungi belonging to the genera *Aspergillus, Fusarium, Mucor,* and *Rhizopus*. Parasites (*e.g., Toxoplasma gondii* and species of *Echinococcus, Cyclospora,* and *Entamoeba*) have also been associated with foodborne diseases^1,3^.

The globalization of the food supply means that populations worldwide are increasingly exposed to new and emerging risks or pathogens^2^. In this context, studies in recent years have hypothesized that ingesting contaminated undercooked meat (with feces or lymph node contents) from cattle and pigs could represent an alternative route of transmission of *Rhodococcus equi* (*R. equi*) for humans, which could partially explain the clinical disease in human patients without a history of contact with livestock and their farm environments^5-8^, a fact that may be considered the main motivation of this study. Considering this scenario, we reviewed the main etioepidemiological aspects of rhodococcosis in livestock and the human relevance of *R. equi* infections in cattle, pigs, and horses, with an emphasis on the virulence of the pathogen related to plasmid patterns.

### Rhodococcosis: general aspects

*R. equi* is the causal agent of rhodococcosis for domestic animals and humans. This opportunistic facultative intracellular bacterium infects phagocytic cells (neutrophils and macrophages) and develops a set of suppurative-to-pyogranulomatous clinical lesions in animals and humans^9^. In recent decades, the virulence of this pathogen in humans and animals has been attributed to plasmids^5,7,8,10^.

*R. equi* is a well-known soil-borne bacterium found in the soil, feces, and organic matter of livestock farms. The domestic animals excrete this microorganism by feces, particularly horses, cattle, and pigs^11,12^.

The inhalation of aerosols from contaminated environments on livestock farms, the ingestion of contaminated food and water, and traumatic percutaneous inoculations are considered the main routes of *R. equi* infection in domestic animals^12-15^.

Equids, particularly horses, represent the main domestic species presenting clinical manifestations of rhodococcosis. Pneumonia, and less frequently non-pulmonary signs, *e.g.*, enteritis, arthritis, and abscesses in organs, constitute the main clinical manifestations of the disease in horses^16^. Among porcine, peccary, and domestic ruminants, infections mainly affect the lymphatic tract, and are commonly identified during slaughter procedures^17,18^. In contrast, clinical disease is rare or sporadic in companion animals and mainly manifests as cutaneous-subcutaneous lesions and pulmonary infections^19,20^.

The routine diagnosis of *R. equi* infections in animals and humans is based on clinical and epidemiological data, microbiological culture, *in vitro* susceptibility testing, imaging techniques, and cytological and histopathological examinations^13-15^. Moreover, molecular methods such as polymerase chain reaction (PCR), sequencing^7,8^, and mass spectrometry^20^ have been increasingly proposed, which have enabled the confirmation of *Rhodococcus* species and the identification of the plasmid virulence pattern of the pathogen^7-9^.

Rifamycin, macrolides, and more recently fluoroquinolones are the main classes of antibiotics to treat rhodococcosis in animals^15,20,21^. In human infections, treatment also includes glycopeptides, carbapenems, beta-lactam derivatives, and sulfonamides^13,14^. Nonetheless, an increase in the multidrug-resistant *R. equi* strains isolated from humans and domestic animals has been described globally and is considered a human health issue^12,21^, including in Brazil^22^.

The general control and prophylaxis of rhodococcosis in livestock and companion animals are based on early diagnosis and measures to minimize the exposure of animals to virulent *R. equi* in the environment (including the management and environmental conditions of breeding farms). In contrast, specific procedures have been targeted exclusively to foals, including the use of hyperimmune plasma and vaccine immunoprophylaxis^23^.

Among humans, rhodococcosis is considered an emerging disease in some countries^24^, with an increasing number of cases reported worldwide^10^. *R. equi* infections occur most frequently in patients who are committed or coinfected by debilitating or immunosuppressive diseases, especially people living with HIV (PLHIV) with advanced immunosuppression (CD4+ T cell count below 200 cells/mm^3^)^5,7,8,25-27^. However, the pathogen can also infect immunocompetent individuals^10,13^.

Severe cavitary pneumonia is the most frequent clinical sign of rhodococcosis in humans and resembles tuberculosis^28^, in addition to other extrapulmonary signs, *e.g.*, progressive weight loss, lymphadenitis, organ abscesses, peritonitis, arthritis, nephritis, osteomyelitis, and meningitis^13,14^.

Historically, the inhalation of aerosols from livestock farms has been the main route of *R. equi* infection in humans^13,14^. Nonetheless, some epidemiological aspects of *R. equi* infections in humans remains unclear as certain patients with rhodococcosis have no history of contact with livestock or the environment of farms, particularly horses, cattle, and pigs^5,7,8^.

## OVERVIEW

### Etiological properties of *R. equi*

*Rhodococcus equi* (also called *Rhodococcus hoagii* and *Prescottella equi*), formerly known as *Corynebacterium equi*, a well-known facultative, versatile, and opportunistic intracellular bacterium taxonomically belongs to the Actinomycetes class and Mycobacteriales order^29^, which includes other pathogenic agents to humans and animals, *e.g., Mycobacteria, Nocardia,* and *Corynebacteria* ^30^. Although more than 40 *Rhodococcus* species are known^31^, *R. equi* has been recognized as the most pathogenic species for humans, livestock, companion animals, and wildlife^11,12,15,32^.

The pathogen presents as small gram-positive cocci or pleomorphic nonmotile, nonspore-forming, and weakly acid-fast bacilli measuring 1-5-µm long. The isolation of bacteria has been carried out on conventional or nonenriched media, such as sheep or bovine blood (5%) or nutrient agar under aerobic conditions from 48 to 72 h^30^. *R. equi* is routinely isolated at 37 °C, although it grows at a wide range of temperatures (10-40 °C). On blood agar media, after 48 h of incubation, colonies measure from 1 to 2 mm in diameter and are typically mucoid (having a capsule), shiny, nonhemolytic, and grayish white. After 72 h, colonies frequently coalesce and develop a typical salmon pigmentation^15^.

NANAT (nalidixic acid, novobiocin, cycloheximide, and potassium tellurite), CAZ-NB (ceftazidime agar, novobiocin, and cycloheximide), TVP (trimethoprim, vancomycin, polymyxin B, and potassium tellurite), and TCP (trimethoprim, cefoperazone, cycloheximide, potassium tellurite, and polymyxin B) are selective media recommended for isolation of *R. equi*, particularly from contaminated material, *e.g.*, soil, feces, manure, and sand^33^.

Among the results of biochemical tests, *R. equi* reveals positive reactions for catalase, lipase, and urease (>18 h) and reduces nitrate to nitrite. In contrast, it is negative for oxidase and neither hydrolyzes esculin nor uses glucose, maltose, or sucrose^15,30,31^. In routine diagnosis, the production of the exoenzymes cholesterol oxidase and phospholipase C by *R. equi* may may also serve for the the phenotypic confirmation of the *R. equi* species that produces a positive Christie, Atkins, Munch-Petersen test with hemolytic *Staphylococcus aureus* ^11,15,30^. Moreover, a *choE*-based PCR assay has also been widely used for species confirmations lately^34^.

### Virulence

Different mechanisms are attributed to the virulence of *R. equi, e.g.*, the presence of an external capsule, the composition of the bacterial cell wall, and the diffusible exoenzymes (cholesterol oxidase and phospholipase C)^9,11,15^. Nonetheless, in last decades, bacterial virulence has been strongly related to the presence of proteins encoded by plasmids^9,16,17,26,35^.

The external capsule and the mycolic acid in the bacterial wall impair the phagocytosis of the pathogen by neutrophils and macrophages. The enzymes cholesterol oxidase and phospholipase C promotes erythrocyte lysis by degrading phospholipids in the erythrocyte membrane, leading to iron release, an essential element for bacterial multiplication and general metabolism^9,11,15^. The production of these exoenzymes can be observed in the CAMP test, which is used in the confirmation of the phenotypic diagnosis of *R. equi* ^11,15^.

### Virulence associated with plasmids

In last decades, the virulence of *R. equi* has been closely related to plasmid-encoded virulence-associated proteins, previously known as virulence-associated protein^5,17,36^ and more recently renamed VAP^9^.

At present, three virulence plasmid types of *R. equi* have been recognized: pVAPA, pVAPB, and pVAPN, which are considered host-^26^ or livestock-adapted. In this context, pVAPA is related to equine isolates^35^, pVAPB is associated with porcine isolates^17^, whereas pVAPN usually occurs in domestic ruminants (bovine and caprine)^37^. In addition to their apparent selectivity for domestic animal species, all the host-associated plasmid types have been identified in infected humans^5,7-10,27,32,38^, supporting evidence of zoonotic behavior in *R. equi* infections^6,7,9^. Moreover, pVAPA and pVAPB isolates show variants or subtypes, with variation in geographic distribution^6,33,35,39,40^. Virulence is intimately related to the ability of *R. equi* to survive in macrophages^9,16^. This overview will adopt the new nomenclature of types (pVAP) and subtypes or variants associated with the virulence of plasmids.

The genes related to the virulence of *R. equi* were initially identified in a pathogenicity island, *i.e., vapA*, *vapB, vapC*, *vapD, vapE*, *vapG*, and *vapH*, in addition to other genes/pseudogenes that were subsequently detected. Nonetheless, the *vapA* gene is likely the most relevant because of its pathogenicity for most domestic animals and humans and its apparent regulatory action of other genes^9,26^. The complex regulation of genes in the pathogenicity of *R. equi* suffers the influence of several factors (including the availability of iron and magnesium) and environmental conditions (*e.g.*, pH and temperature), which can influence gene expression. Soil pH and environment temperature may contribute to the endemicity of rhodococcosis in some regions, countries, and farms^9,15,16,26^.

### pVAPA type

Among the pVAPA-type isolates (formerly virulent strains or VapA), circular large plasmids (85-90 kilobases or kb) contain genes related to the expression of proteins (antigens) of 15-17 kDa. Since 1991, pVAPA-type strains have been found in diseased foals (equine-type)^36^. pVAPA isolates predominantly cause severe suppurative bronchopneumonia in foals, as well as colitis and mesenteric lymphadenitis^15,35^. This host-adapted type of *R. equi* also infects humans, mainly PLHIV^7,10,25^, including in Brazil^27^. It has been proposed that *R. equi* harboring pVAPA-type isolates can remain viable inside macrophages and neutrophils and resist phagocytosis, favoring severe and chronic infections^9,26^.

To date, 14 distinct variants or subtypes of pVAPA are known. They are subdivided as follows: 52-, 85- (subtypes or variants I, II, III, IV, and V), 87- (I, II, and III), and 90-kb (I, II, III, IV, and V). Among *R. equi* strains isolated from horses bred in the Americas, Europe, and Australia, a predominance of 85- and 87-kb subtype I has been reported^40,41^, in addition to a minor occurrence of 87-kb subtype III in Brazil^40^. Isolates containing the 85-kb subtype II are predominant in France. In the USA, 85-kb subtypes III and IV have been found. In turn, strains harboring 87-kb subtype II and 90-kb subtypes I, II, III, IV, and V plasmids have been described in Japan^35,40^.

In 2022, a novel 52-kb variant was identified in a foal with clinical signs of rhodococcosis from the Netherlands^42^. The genome sequences of the 14 representative plasmid DNAs of the pVAPA variants (subtypes) showed that the five variants of 85-kb plasmids are intimately related and that five 90-kb variants are closely associated. In contrast, 87-kb subtype I and subtype III are similar and relatively distinct from 87-kb variant II^35^.

### pVAPB type

Isolates of the *R. equi* pVAPB type (formerly intermediate virulence strains or VapB) possess circular large plasmids (79 to 100 kb) that contain genes that express 20 kDa proteins^17^. Since 1995, isolates of the pVAPB type have been identified in the lymph nodes with and without lesions among domestic pigs and wild boars (porcine-type)^17,39,43,44^, from the feces of pigs^13^, and from human patients affected by immunosuppressive conditions/diseases, particularly PLHIV, in Thailand^5^, Brazil^8,27^, Japan^7^, and Cuba^10^.

More than 20 subtypes (or variants) have been recognized in pVAPB-type strains (represented by cardinal numbers)^6,15,17^. A tendency toward the geographic identification of subtypes or variants of the *R. equi* pVAPB type in pigs and wild boars has been observed, with a predominance of subtypes 1 and 2 in Asia^5,17,45,46^, subtype 5 in Europe^6,39,47^, and subtype 8 in South America, particularly Brazil^44^.

Sporadically, pigs^17^ and wild boars^45^ may harbor pVAPA strains, whereas pVAPB may occur in cattle^6^.

### pVAPN type

In 2015, a third or novel type of plasmid associated with the virulence of *R. equi*, called pVAPN (N for either no-A or no-B), was proposed. In contrast to the circular pVAPA and pVAPB types^9^, the pVAPN plasmids (120 to 125 kb) are linear^37,38^. The pVAPN type has been mainly associated with bovine or caprine isolates (bovine or ruminant-type), causing pyogranulomatous lesions in the lungs, liver, spleen, and lymph nodes^8,37,48,49^. Sporadically, reports have also identified the *R. equi* pVAPN type in diseased companion animals^19^. As pVAPA and pVAPB isolates, human infections caused by *R. equi* harboring pVAPN have also been associated with immunosuppressive disorders (mainly HIV advanced disease), as reported in Brazil^8^, the USA^32^, Japan^7^, and Cuba^10^.

### Avirulent or plasmidless

These *R. equi* strains are called avirulent or plasmidless because they lack plasmids containing genes related to the three known host-adapted virulence types (*i.e.*, pVAPA, pVAPB, and pVAPN)^9,16^, although these isolates could hypothetically carry other unidentified virulence-associated plasmids^8^. Avirulent isolates have been recovered from the soil surface and feces of livestock breeding farms or their environments (mainly horses, cattle, and pigs)^15^. Apparently, avirulent or plasmidless isolates are unable to induce clinical disease in domestic and wildlife animals, although they may be isolated from apparently healthy lymph nodes of livestock^6,8^.

Plasmidless isolates have also been described in the environment of human recreational areas (*e.g.*, the soil and sand of parks and yards)^50^. Additionally, avirulent strains have been identified in humans with rhodococcosis with and without immunosuppressive conditions^5,7,8,10,27^.

### Epidemiology of livestock infections

Magnusson first described rhodococcosis in domestic animals in 1923 in Sweden based on cases of pyogranulomatous pneumonia in foals^9,16^. The disease has a worldwide distribution, with a certain predominance in countries with temperate climates^9,16,51^.

*R. equi* is a soilborne bacterium that actively multiplies in the environment of livestock animals, particularly horses, cattle, and pig breeding farms. The organism requires minimal nutritional temperature and humidity conditions, and is commonly found in soil, manure, and herbivore feces^11,12^. The microorganism multiplies over a wide range of temperatures (15 to 40°C) but mainly during warmer periods (early to late summer) of the year, which likely contributes to the high prevalence of rhodococcosis in tropical regions and countries^15^.

The pathogen is widely found on the soil surface of livestock farms^12^. The multiplication of the pathogen is favored in soils with warm temperatures and pH values close to neutral, which likely limits the occurrence of rhodococcosis in cold-climate countries or breeding farms with acidic soils^51^. Seasonal variations among clinical infections in horses can be attributed to oscillations in climatic conditions that can contribute to bacterial multiplication in the environment^11,15^.

*R. equi* is highly resistant to desiccation and exposure to sunlight and remains viable for >12 months in farm facilities and soil^15^. Moreover, an excess of feces in dry environments contributes to the formation of aerosols containing *R. equi* ^23^.

Although *R. equi* is not considered part of the normal enteric microbiota of animals and humans^15^, it is frequently isolated from the feces of a wide variety of herbivores and omnivores, *e.g.*, horses, pigs, cattle, sheep, and goats^11,12^.

### Horses

Particularly in foals, intestinal colonization occurs early, with the microorganism detectable in feces as soon as five days of age. The microorganisms actively multiply in the intestine of foals around the third month of age and subsequently decline^15^. In adult horses, *R. equi* is also isolated from feces^11^. Foals can eliminate virulent pVAPA *R. equi* strains via feces^41^, which contributes to the infection of other foals, other domestic animals, and humans and to the contamination of the environment and the maintenance of the pathogen in the soil of breeding farms^52^. Moreover, coprophagy in foals^53^ may favor oral infection by pVAPA strains from feces.

*R. equi* can also be found in the upper respiratory tract of foals and adult horses without pulmonary signs (subclinical infections), particularly on farms with excessive contaminated soil^23,41^. Regarding this, a study investigating the prevalence of *R. equi* in the nasal cavity of 1,010 apparently healthy horses from 341 nonendemic farms in Brazil found that approximately 1% of the animals tested positive and that 3% of the farms had at least one positive horse in which the microorganism was isolated^54^.

Aerosolized particles of *R. equi*, which are suspended in excessively dry environments, are inhaled by foals and encompass the main route of transmission of the pathogen for equids^23^. The rate and density of tracheal colonization of *R. equi* in foals differed from their birth month and gradually increased according to the months with the rise in outside temperature in Japan. The environmental conditions of horse-breeding farms play a vital role in determining the prevalence and severity of *R. equi* infection in foals^55^. Aerial particles can also contaminate the food and water of animals, predisposing them to infection via oral route^15^.

Globally, horses are the main livestock animals susceptible to *R. equi* infections^23^. Nonetheless, an emergence^24^ and an increase in clinical cases have been reported in humans^10,13^ and companion animals^19^. Among domestic ruminants (cattle, buffaloes, sheep, and goats) and pigs, clinical disease is unusual^11,15^. Nonetheless, *R. equi* has been frequently isolated from the lymph nodes of slaughtered cattle^8^, pigs^17,39,47^, and wild boars^43-45^ with and without lymphadenitis^18^.

Clinical disease commonly occurs in foals that are aged from two weeks to six months^11,12,23^, with a predominance of pneumonic cases occurring from approximately one to three months^15,40^. During this period, the high occurrence of clinical disease is attributed to the failure of passive immunity acquired by ingesting colostrum and the relative immaturity of the immune system of young foals. Conversely, rhodococcosis is a rare clinical condition in adult horses that manifests similar signs to those in foals and is related to coinfections with microorganisms that induce immunosuppressive disorders in susceptible hosts^11,15^.

Some deficiencies in animal management practices and environmental or underlying conditions have been considered risk factors for equine rhodococcosis in endemic farms, including insufficient ingestion of colostrum by foals, inadequate removal of feces and organic matter from facilities, excessive dust, proximity of stables and paddocks of categories of equids of different ages, high turnover of horses, overpopulation of foals and mares, dirty pastures, dry climates, and high temperatures^11,12,15,23^.

The morbidity rates of the disease can vary widely and annually among horses, individual farms, and geographic regions, affecting 30% or more of the animals on endemic farms. Nonetheless, the prevalence of equine subclinical infections is typically >90%. Its mortality rate may be extremely high (up to 30%), especially in cases of late diagnosis and/or treatment approaches^15,23^.

### Porcine

Porcine are considered the second most affected livestock by *R. equi* ^18^. Among pigs, wild boars, and peccaries, the pathogen predominantly infects the lymphatic tract, causing lymphadenitis^17,39,43,45,47^, although the microorganism can be isolated from apparently normal lymph nodes^6,17,18,39,44-46^.

The transmission mechanisms related to pigs, wild boars, and peccary species are yet to be fully understood. It has been suggested that transmission of the pathogen occurs mainly via the oral route by the consumption of contaminated water and food. Nonetheless, the gross appearance of *R. equi* lymphadenitis in pigs, wild boars, and peccary species is indistinguishable from that induced by other bacteria that also cause lymphadenitis or tuberculoid lesions — which are represented mainly by mycobacteria, other actinomycetes (*Trueperella pyogenes* and *Nocardia* and *Corynebacterium* species), staphylococci, streptococci, and enterobacteria^15,18^.

Lara *et al*.^18^ investigated the frequency of bacteria in 378 lymph nodes with and without lymphadenitis of slaughtered pigs and wild boars from the central region of the Sao Paulo State, Brazil. *M. avium* (types 1 and 2) and *R. equi* constituted the prevalent agents in the lymph nodes with tuberculoid lesions and without lymphadenitis, followed by *Trueperella pyogenes*, streptococci, staphylococci, corynebacteria, and enterobacteria species.

### Domestic ruminants

Rhodococcosis is an uncommon disease in cattle, buffaloes, goats, and sheep. As in pigs, the transmission of the pathogen to domestic ruminants is yet to be fully understood. Apparently, the ingestion of contaminated water and pasture and the inhalation of aerosols represent probable routes of the transmission of the pathogen because the microorganism has been isolated from the lymphatic and respiratory tract of naturally infected animals of this ruminant species^8,11,56^.

### Molecular epidemiology of VAP types in host-adapted livestock species

The use of molecular techniques, particularly PCR and sequencing methods, has advanced diagnosis. Epidemiological and virulence studies have also indicated the probable origin of virulent types of *R. equi* in diseased animals and humans based on host-adapted livestock^7-9,16,26,35^. Table 1 summarizes some studies that investigated the virulence plasmid profile of *R. equi* in livestock and wild boars intended for human consumption and human infections.

**Table 1:** Selected studies involving virulence plasmid patterns of *Rhodococcus equi* in livestock and wild boars intended for consumption and human infections

Species		Samples (N samples)	Number of *R. equi* isolated (%)	VAP types and subtypes-positive isolates			Plasmidless	Article and country
				pVAPA and subtypes	pVAPB and subtypes	pVAPN		
Humans								
		bronchial wash, nasal mucosa, blood, lung, hepatic, and cutaneous fragments and the fingernail region (N=20)	20 isolates (stocked)	pVAPA: 4 isolates 85-kb subtype I and 87-kb subtype I	pVAPB: 5 isolates Subtype 8	NI	11 isolates	Ribeiro *et al*.^27^ Brazil
		different clinical samples (N=38)	39 isolates (stocked)	pVAPA: 8 isolates 85-kb subtype I	pVAPB: 10 isolates Subtypes: 1, 10, 22, 26, 31, and 32	pVAPN: 7 isolates	13 isolates (1 isolate excluded)	Takai *et al.* ^7^ Japan
		data not available (N=26)	26 isolates (stocked)	pVAPA: 4 isolates	pVAPB: 6 isolates	pVAPN: 1 isolate	14 isolates (1 isolate nontippable)	Salazar-Rodriguez *et al.* ^10^ Cuba
Porcine								
	pigs	submaxillary lymph nodes without lymphadenitis (N=1,832)	56 isolates (3.1%)	pVAPA: 1 isolate 90-kb	pVAPB: 54 isolates Subtypes: 1, 2, 3, 4, and 5	NI	1 isolate	Takai *et al.* ^17^ Japan
	pigs	submaxillary lymph nodes without lymphadenitis (N=1,173)	164 isolates (14%)	none	pVAPB: 44 isolates Subtypes: 1, 4, 5, 6, 7, and 1 new variant	NI	120 isolates	Makrai *et al.* ^39^ Hungary
	wild boars (*Sus scrofa*)	submaxillary lymph nodes (N=482)	82 isolates from 60 samples (12.4%)	none	pVAPB: 21 isolates Subtypes: 1, 5, 21, and 3 new variants (25, 26, and 27)	NI	61 isolates	Makrai *et al.* ^43^ Hungary
	pigs	submaxillary and mesenteric lymph nodes with (N=129) and without lymphadenitis (N=129)	19 isolates: 17 isolates with lymphadenitis and 2 without lymphadenitis (7.4%)	none	pVAPB: 12 isolates Subtypes: 1, 8, 10, and 29	NI	7 isolates	Ribeiro *et al.* ^44^ Brazil
	wild boars (*Sus scrofa*)	submaxillary and mesenteric lymph nodes with (N=60) and without lymphadenitis (N=60)	4 isolates with lymphadenitis (6.7%)	none	pVAPB: 4 isolates Subtype: 8	NI	none	
	wild boars (*Sus scrofa*)	submaxillary lymph nodes (N=86)	45 isolates (52%)	pVAPA: 1 isolate 87-kb subtype II	pVAPB: 21 isolates Subtypes: 1, 2, 4, and 3 new variants	NI	23 isolates	Sakai *et al.* ^45^ Japan
	pigs	submaxillary lymph nodes with lymphadenitis (N=232)	57 isolates (24.6%)	none	pVAPB: 56 isolates Subtypes: 1, 2, 3, 9, 13, and 14	NI	1 isolate	Matsuoka *et al.* ^46^ Japan
		submaxillary lymph nodes without lymphadenitis (N=420)	10 isolates (2.4%)	none	pVAPB: 6 isolates Subtypes: included above among lymph nodes with lesions	NI	4 isolates	
Miscellaneous*								
	humans	sputum, blood, abscesses, bronchoalveolar lavage, and pleural fluids (N=69)	69 isolates (stocked)	none	pVAPB: 52 isolates Subtypes: 1, 4, 7, 12, and 4 new variants	NI	17 isolates	Takai *et al.* ^5^ Thailand
	pigs	submaxillary lymph nodes with lymphadenitis (N=500)	20 isolates from 4 positive-samples (0.8%)	none	pVAPB: 15 isolates Subtypes: 1, 7, 13, and 15	NI	5 isolates	
		submaxillary lymph nodes (N=398)	105 isolates (26.6%)	none	pVAPB: 103 isolates Subtypes: 1, 4, 5, 6, 7, 10, 11, 21, and 31	NI	2 isolates	Witkowski *et al.* ^6^ Poland
	bovines	submaxillary lymph nodes (N=234)	3 isolates (1.3%)	none	pVAPB: 3 isolates Subtype: 26	NI	none	
	horses	retropharyngeal and tracheobronchial lymph nodes (N=198)	1 isolate (0.5%)	pVAPA: 1 isolate 85-kb subtype I	none	NI	none	
	humans	bronchial wash, blood, abscesses, skin lesions, and organ fragments (N=74)	74 isolates (stocked)	none	pVAPB: 7 isolates Subtypes: 8 and 11	pVAPN: 2 isolates	65 isolates	Ribeiro *et al.* ^8^ Brazil
	bovines	submaxillary, mesenteric, and mediastinal lymph nodes with lymphadenitis (N=100)	31 isolates (31%)	none	none	pVAPN: 13 isolates	18 isolates	
		feces (N=100)	49 isolates (49%)	none	none	none	49 isolates	

Miscellaneous

* = comparative studies between humans, bovines, pigs, or horses; N = number; % = percentage; NI = not investigated.

### Horses

In Brazil, Ribeiro *et al*.^40^ have investigated for the first time the virulence associated with plasmids among 41 *R. equi* strains that had been isolated from 40 lungs and one bronchial washing of foals in the Sao Paulo State from 1991 to 2003. Of the isolates harboring exclusively the pVAPA type, the authors found 33 *R. equi* isolates as the 87-kb subtype I and six, as the 85-kb subtype I, proposing two isolates as a new variant (87-kb subtype III).

A plasmid virulence investigation of 57 pVAPA-positive *R. equi* strains recovered from pneumonic foals or during necropsy in 13 horse breeding farms from Poland in 2017 identified 48 isolates harboring 85-kb subtype I (82.8%), eight carrying 87-kb subtype I (13.8%), and one isolate (1.7%) with a unique restriction cleavage, designated as a new variant of pVAPA, 85-kb subtype V^57^.

In Germany, the presence of the pVAPA type was investigated in 37 and 55 *R. equi* strains isolated from nasal swabs and tracheal samples of foals, showing that 68 and 73% were VAPA-positive, respectively. Moreover, in 64 randomly selected (from March and July, 2003) soil samples from paddocks and stables of equine farms, 21 (3/14) and 81% (13/16) were VAPA-positive, highlighting the identification of this virulence type in the nasal and tracheal microbiota of foals and the soil of horse breeding farms^41^.

### Pigs, wild boars, and peccary

In Japan, Takai *et al*.^17^ first described the predominance of pVAPB of *R. equi* in the apparently healthy lymph nodes of pigs and the similarity of virulence profiles between pig and human isolates. In Hungary, Makrai *et al*.^39^ investigated the virulence profile of 164 *R. equi*, isolated from the submaxillary lymph nodes of pigs slaughtered without macroscopic lesions, found pVAPB subtypes 1, 4, 5, 6, and 7, and a new variant. The same research group in Hungary detected subtypes 1, 5, 21, and three new variants (25, 26, and 27) of the *R. equi* pVAPB type, isolated from the 60 submaxillary lymph nodes of wild boars slaughtered without apparent lesions^43^.

In Brazil, a similar study in the central region of the Sao Paulo State, Brazil, focused on the virulence profile of *R. equi* isolated from the lymph nodes of 19 slaughtered pigs (17 with and two without lesions) and four wild boars with lesions intended for human consumption, finding pVAPB subtypes 1, 8, 10, and 29, with a predominance of subtype 8 in both species^44^.

Sakai *et al*.^45^, in Japan, isolated *R. equi* harboring pVAPB of subtypes 1, 2, 4 and three new variants from submandibular lymph nodes without apparent lesions of 86 wild boars.

A study in Poland investigating the plasmid virulence profile of *R. equi* from submaxillary lymph nodes in apparently healthy 23 wild boars, two red deer (*Cervus elaphus*), and two roe deer (*Capreolus capreolus*), obtained during hunting, found 16 isolates from wild boars that were pVAPB-positive (subtypes 5, 7, and 11), whereas the remaining strains from wild boars and deer were avirulent^58^. Another study in Poland investigated virulence related to the plasmids in *R. equi* that had been isolated from 1,028 lymph nodes from apparently healthy slaughtered pigs, bovines, and horses. The authors isolated *R. equi* from 26.6% (105/395) of the pigs, 1.3% (3/234) of the submaxillary lymph nodes of cattle, and from only 0.5% (1/198) of the tracheobronchial lymph node of a horse. Among all lymph nodes, purulent lesions only occurred in 0.8 % of the swine submaxillary lymph nodes samples (3/398). The authors found *R. equi* in two of them. Of the 107 *R. equi* strains isolated from pigs, the authors predominantly found *R. equi* pVAPB subtype 5, followed by subtypes 1, 4, 6, 7, 10, 11, 21, and 31 with a minor occurrence^6^.

Most recently, Matsuoka *et al*.^46^ described the predominance of the pVAPB type (subtypes 1, 2, 3, 9, 13, and 14) in 57 *R. equi* strains isolated from macroscopically detectable lesions in the submaxillary lymph nodes of 232 growing-finishing pigs slaughtered in Japan.

### Bovines

A study in Brazil investigated the three host-associated virulence plasmid types in 31 *R. equi* strains isolated from lymphadenitis and 49 isolates of non-diarrheic fecal samples from slaughtered cattle. The plasmidial pattern showed that, among the cattle lymph nodes with macroscopic lesions, 41.9% of the isolates were of the pVAPN type. The authors found no virulent type in the isolates from feces, highlighting the high occurrence of pVAPN in the lymph nodes of slaughtered cattle^8^.

### Goats

Six cases of rhodococcosis in goats in the USA showed bronchopneumonia, pulmonary abscesses or caseous lesions in lungs (3/6=50%), caseous lesions in visceral and abdominal lymph nodes (2/6=33.3%), osteomyelitis (2/6=33.3%), subcutaneous abscesses (1/6=16.7%), and caseous lesions in spleen, liver, and muscle (1/6=16.7%), which enabled the isolation of eight *R. equi* strains. Virulence-associated plasmid pattern analysis showed that 87% of these isolates harbor the pVAPN type^56^.

Recently, a study in Japan carried out an experimental infection of goats by the intravenous administration of *R. equi* harboring the pVAPN type, finding a latent infection of the lymph nodes despite no significant clinical manifestations in the studied animals^49^.

### Human implications

The first case of human rhodococcosis was described in a man from the USA in 1967. He presented lung abscesses, an impaired immune response, and prolonged corticoid therapy owing to chronic hepatitis. The first case of a human patient coinfected with HIV and *R. equi* was described in a man with lung abscess and persistent bacteremia in 1986^31^. Over the subsequent 15 years, *R. equi* infections in humans were considered rare, with approximately 15 reported cases^59^. Conversely, since 1986, an increasing number of *R. equi* infections in humans has been described, particularly related to severe immunosuppression, including post-transplant immunosuppressive therapy and, most notably, PLHIV with advanced disease and, to date, rhodococcosis is considered an emerging disease^5,7,8,10,24,26,27^.

In 2000, *R. equi* infection was described in a PLHIV with pulmonary signs. It may be considered the first case report of rhodococcosis in Brazil^60^.

In general, rhodococcosis is an occupational disease that affects mainly men aged from 30 to 50 years. *R. equi* infections are intimately related to certain groups of vulnerability, especially immunosuppressed patients with a set of underlying diseases, including individuals with nephropathies, hepatopathies, neoplasia, alcohol abuse, transplants, those treated for prolonged periods with immunosuppressive drugs, and, most frequently, PLHIV^11,27,59^. Nonetheless, *R. equi* infections have also been reported in immunocompetent humans^10,13^ and in individuals without HIV-infection^5^, including in Brazil^27^.

Since the first case reported in humans, the main routes of transmission of the pathogen have been considered the inhalation of aerosol particles containing *R. equi* in the farm environment (soil, feces, manure) of livestock^11,13,14^ and the traumatic percutaneous inoculation of the bacterium^15^.

Early studies of human rhodococcosis have focused on the association between the underlying conditions of patients and their history of contact with livestock and companion animals. In this context, a study in the USA investigated 12 cases of human rhodococcosis (six of which were PLHIV), of whom two patients had a history of contact with horses, one with a dog and the other with a farm environment^59^. However, to date, the real impact of the transmission of *R. equi* from domestic animals-to-humans is yet to be fully understood^16^ since the source of infection in many cases is unknown, and some patients lack a history of contact with farm environments or domestic animals^27^.

Chronic cavitary pneumonia (with pleural effusion), cough, and fever constitute the main clinical signs in humans infected with *R. equi* ^13,14,16^, resembling mycobacterial infections^28^.

Extrapulmonary clinical presentations are less common. They include diarrhea, cachexia, hepatic disorders, organ abscesses, nephropathies, pleurisy, peritonitis, and septic arthritis^13,14,59^.

The routine diagnosis of human infections resembles that of animal infections. It is based on clinical and epidemiological data, microbiological culture, imaging techniques, and cytological and histopathological examinations^13,14,16^. However, diagnosis based on molecular methods have been increasingly encouraged^7,9^.

Rifamycin, macrolides, fluoroquinolones, glycopeptides, carbapenems, beta-lactam derivatives, and sulfonamides constitute the main groups of antibiotics to treat human infections^13,14^. However, multidrug-resistant *R. equi* strains isolated from humans have been increasingly described worldwide. They are considered a human health concern^14^, including in Brazil^22^. Patients receive treatment for several weeks until the complete remission of signs. Relapses and clinical complications are common. Rhodococcosis cause concern for death in PLHIV^14^.

No specific measures are recommended to prevent/control this disease in humans. *R. equi* affects a variety of domestic species. It widely occurs in farms (particularly those with horses and ruminants) and in the soil and sand of human entertainment parks and squares. Therefore, close contact between human patients with any immunosuppression conditions (especially PLHIV) and diseased livestock or companion animals presenting compatible signs of *R. equi* infections, along with the environment of farms, is not recommended. Additionally, the consumption of raw or undercooked bovine, porcine or equine products and derivatives from slaughtered animals without food inspection should be avoided^8,15,27^.

### PVAP types in humans

In 1991, pVAPA-type strains were identified in foals from Japan as having a virulence plasmid profile in animals presenting severe suppurative bronchopneumonia, colitis, and mesenteric lymphadenitis^36^. Since then, foals have been related as the primary origin of human infections by *R. equi* ^11,13,14^. The presence of human patients infected with pVAPA could explain the zoonotic behavior of the pathogen and its transmission from equine to humans^9^. Nonetheless, zoonotic transmission from animals-to-humans is yet to be fully understood because some diseased patients have no history of close contact with livestock or their farm environments^27^. Table 1 summarizes some studies that investigated the virulence plasmid profile of *R. equi* in human infections and in livestock and wild boars intended for human consumption.

Subsequently the identification of pVAPA in foals 36, the same research group in Japan investigated the virulence plasmid types of *R. equi* isolated from the lymph nodes of pigs and described the predominance of pVAPB and the similarity of plasmidial virulence between pigs and humans^17^. In this context, from 1993 to 2001, Takai *et al*.^5^ compared the virulence plasmid profiles of 69 cases of rhodococcosis in humans (of which 50 patients were PLHIV) and 20 *R. equi* strains isolated from pigs in Thailand, where porcine consumption by humans is greater than bovine. Of the studied humans, 52 (75.3%) isolates harbored pVAPB-type plasmids containing subtypes 1, 4, 7, 12, and four new variants, with a predominance of type 4, whereas 17 (24.7%) isolates were avirulent. Of 50 (72.5%) PLHIV, 37 (74%) patients carried the pVAPB type, reinforcing the association of rhodococcosis and HIV advanced disease^5,7,26,27^. In pigs, a predominance of pVAPB isolates subtypes 1, 7, 13, and 15 has been observed, indicating an identity of virulent type and similarity between subtypes of *R. equi* detected in humans and pigs in Thailand. These findings indicate a probable route of the transmission of the pathogen from pigs to humans via the consumption of raw or improperly cooked pork^6,15^.

A study in Brazil investigated the virulence profiles of two asymptomatic individuals and 18 diseased humans infected with *R. equi* (of which nine were PLHIV) from the states of Sao Paulo, Rio Grande do Sul, Rio de Janeiro, and Minas Gerais. Among the 20 humans, 11 had a history of contact with livestock (cattle and horses) or farm environments, whereas none reported contact with pigs or pig breeding environment. The plasmid virulence pattern showed five individuals harboring pVAPB-type isolates, four harboring pVAPA-type isolates, and 11 plasmidless isolates. Among the humans infected with *R. equi* pVAPA, three were 87-kb subtype I and one 85-kb subtype I^27^, a result that coincides with a previous plasmid virulence in foals from the states of Sao Paulo and Rio Grande do Sul, Brazil^40^. In total, two patients with pVAPA-type strains had contact with horses, reinforcing the zoonotic risk of human infection by the equine-type of *R. equi*. All five *R. equi* pVAPB type isolates were detected among PLHIV, harboring exclusively subtype 8, the same plasmid profile of *R. equi* that had been identified as predominant by the same research group in Brazil from the lymph nodes of pigs and wild boars intended for human consumption in the Sao Paulo State^44^, highlighting the association of rhodococcosis and advanced HIV and a probable infection of humans by the consumption of undercooked or raw meat from pigs^5,15^.

The comparative virulence plasmid patterns of 31 *R. equi* strains isolated from bovine lymphadenitis, 49 from the feces of slaughtered cattle, and 74 from diseased humans with rhodococcosis in Brazil showed seven pVAPB-type (subtypes 8 and 11) and two pVAPN-type isolates in humans, all of which were isolated from lung samples from PLHIV. Among the lymph nodes of cattle, 13 (41.9%) isolates were of the pVAPN type^8^. Thus, the identification of the *R. equi* pVAPN type in humans with rhodococcosis and the high frequency of *R. equi* harboring virulent pVAPN in the lymph nodes of slaughtered cattle could represent an emergent risk to humans in some countries^7,10,24,32,38^, particularly Brazil^8^, via the probable ingestion of contaminated raw or undercooked beef products.

In Japan, Takai *et al.* ^7^ investigated the virulence associated with plasmids of 39 previously reported *R. equi* strains isolated from people living with or without HIV. That study found pVAPA, pVAPB, and pVAPN in eight (20.5%), 10 (25.6%), and seven (17.9%) *R. equi* strains, respectively. Of the 29 *R. equi* isolates from PLHIV, seven (24.1%), 10 (34.5%), and five (17.2%) carried pVAPA, pVAPB, and pVAPN types, respectively. These findings reinforce the high frequency of PLHIV in advanced disease with rhodococcosis and indicate that the identification of *R. equi* harboring host-adapted pVAPB (porcine-type) and pVAPN (bovine-type) plasmid types could partially explain the disease in humans without a history of contact with pigs, bovines, or farms. Moreover, the likely infection of humans by *R. equi* by the ingestion of contaminated raw or undercooked meat from cattle, pigs, and, occasionally, horses, could represent a presumable foodborne transmission of this bacterium^5-9,27^.

A case-series study of plasmid virulence profile of *R. equi* was conducted in 26 PLHIV from Cuba. The virulence analysis of the *R. equi* isolates showed 4, 6 and 1 isolates harboring pVAPA, pVAPB, and pVAPN livestock-adapted types, respectively^10^, suggesting a probable transmission of the pathogen from horses, pigs, and domestic ruminants for humans^5,7-9,27^, respectively, and highlighting the emergence of human rhodococcosis in some countries^24^.

Another hypothesis of *R. equi* transmission from pigs or cattle to humans involves the consumption of uninspected slaughtered pigs or cattle with cross-contamination of meat with feces or lymph node contents since, of these livestock species, *R. equi* infection is practically restricted to the lymphatic tract. The pathogen has been identified in feces^8^, including virulent types^33^.

Studies have also described that diseased humans worldwide are infected with avirulent *R. equi* with and without immunosuppressive or debilitated diseases^5,7,8,10,27^. Plasmidless isolates widely occur in the soil of farms and in parks and yards for human entertainment. In these cases, the presence of a capsule and mycolic acid-containing lipid-rich cell wall structure, the production of exoenzymes (phospholipase C and cholesterol oxidase), the presence of multidrug-resistant isolates, and pyogranulomatous reactions induced by the agent could suffice to determine the pathogenicity of the plasmidless *R. equi* isolates^15^. Alternatively, these patients could be infected by other virulent plasmid types that are yet to be identified^8^.

Despite circumstantial evidence of a probable transmission of virulent *R. equi* strains for humans by ingestion of contaminated meat from livestock slaughtered, this route of transmission remains a hypothesis that requires investigation/confirmation in further comprehensive studies.

## CONCLUSION

The identification of horses, pigs, and cattle/goats as host-adapted livestock species of pVAPA, pVAPB, and pVAPN virulence plasmid types, respectively, indicate that *R. equi* infections in humans are presumably zoonotically acquired and, in addition to the direct transmission of the pathogen to humans by close contact with horses and their environment (related to pVAPA or equine-type), these bacteria could be transmitted to humans by the consumption of contaminated undercooked or raw meat from pigs (pVAPB or porcine-type) and cattle (pVAPN or ruminant-type), representing a probable foodborne pathogen.

## Data Availability

The complete anonymized dataset supporting the findings of this study is included within the article itself.
